# The Nuclear Localization of γ-Tubulin Is Regulated by SadB-mediated Phosphorylation*[Fn FN2]

**DOI:** 10.1074/jbc.M114.562389

**Published:** 2014-06-18

**Authors:** Greta Eklund, Stefan Lang, Johan Glindre, Åsa Ehlén, Maria Alvarado-Kristensson

**Affiliations:** From the Division of Molecular Pathology, Department of Laboratory Medicine, Lund University, Skåne University Hospital, SE-20502 Malmö, Sweden

**Keywords:** Cell Cycle, Centrosome, Nucleus, Phosphorylation, Tubulin

## Abstract

γ-Tubulin is an important cell division regulator that arranges microtubule assembly and mitotic spindle formation. Cytosolic γ-tubulin nucleates α- and β-tubulin in a growing microtubule by forming the ring-shaped protein complex γTuRC. Nuclear γ-tubulin also regulates S-phase progression by moderating the activities of E2 promoter-binding factors. The mechanism that regulates localization of γ-tubulin is currently unknown. Here, we demonstrate that the human Ser/Thr kinase SadB short localizes to chromatin and centrosomes. We found that SadB-mediated phosphorylation of γ-tubulin on Ser^385^ formed chromatin-associated γ-tubulin complexes that moderate gene expression. In this way, the C-terminal region of γ-tubulin regulates S-phase progression. In addition, chromatin levels of γ-tubulin were decreased by the reduction of SadB levels or expression of a non-phosphorylatable Ala^385^-γ-tubulin but were enhanced by expression of SadB, wild-type γ-tubulin, or a phosphomimetic Asp^385^-γ-tubulin mutant. Our results demonstrate that SadB kinases regulate the cellular localization of γ-tubulin and thereby control S-phase progression.

## Teaser

**Background:** γ-Tubulin moderates gene expression by accumulating in the nucleus during early cell division.

**Results:** SadB-mediated phosphorylation of Ser^385^-γ-tubulin exposes the nuclear localization signal of γ-tubulin.

**Conclusion:** SadB kinases regulate the cellular location of γ-tubulin and in this way control cell growth.

**Significance:** All knowledge on tubulins can aid the design of more efficient chemotherapeutic agents.

## Introduction

During symmetric cell division, a cell becomes two identical daughter cells in a process that is highly regulated. An important regulator of cell division is γ-tubulin, which orchestrates microtubule assembly and ubiquitin-mediated processes during cell growth (1–7). In the cytosol, γ-tubulin is part of a ring-shaped protein complex called γTuRC (1, 4–7). Recently, a bipartite nuclear localization signal (NLS)^2^ that assists translocation of γ-tubulin to the nucleus was identified in the C terminus of this protein (8). In the nucleus, γ-tubulin regulates E2 promoter-binding factor (E2F) transcriptional activity (8–11), ensuring a transient transcription of genes necessary for centrosomal duplication and DNA-replication (8, 9). Moreover, nuclear γ-tubulin also forms complexes with Rad51 and C53 (10, 11).

As of yet, little is known about cellular signal pathways that regulate the localization of γ-tubulin. Various studies demonstrate that phosphorylation of γ-tubulin regulates its activity and function (12–14). However, upon phosphorylation of Ser^131^ in cytosolic γ-tubulin by SADB kinases (mouse SADB, human SAD1 (hSAD1)/BRSK1), γ-tubulin regulates centrosomal duplication and function (2, 12). SADBs are serine/threonine kinases involved in cell cycle progression (2). Three different mouse isoforms designated SADB_L_ (long), SADB_S_ (short), and SADB_S1_ (2) and a human long isoform called Sad1 (SadB_L_) have been described (15). The actions of SADB kinases oscillate during the cell cycle and are most pronounced in advanced G_1_, G_1_-S, and S phases (2, 16). These fluctuations are necessary for cell cycle progression, because SADB kinases control Cdk1 activity (15) and centrosome biogenesis (2, 16).

Nuclear γ-tubulin is necessary for S phase execution, but the mechanism(s) that regulates its nuclear accumulation remains elusive. In the present study, we found that there are two pools of γ-tubulin, one cytosolic and the other nuclear. Using mammalian cell lines (8), we demonstrate that only the cytosolic γ-tubulin pool contains the γTuRC components γ-tubulin complex protein 2 (GCP2) and GCP3. Furthermore, in U2OS and NIH3T3 cells, the size of the nuclear γ-tubulin pool varied, depending on the levels of SadB kinases, and our results show that SadB-mediated phosphorylation of Ser^385^ in γ-tubulin regulates the localization and function of γ-tubulin.

## EXPERIMENTAL PROCEDURES

### 

#### 

##### cDNA and Reagents

Human C-terminal GFP-tagged γ-tubulin/pcDNA3 was provided by Dr. J. Bartek (17), pcDNA3-hemagglutinin (HA)hE2F1 was furnished by Dr. J. Nevins (18), pGL3-TATA-6xE2F-Luc was from Dr. K. Helin (19), and the cDNA encoding mouse SADB_L_ (pcDNA3-FLAG-SADB_L_) was a gift from Dr. J. Sanes (20). *SADB* shRNA, *SadB* shRNA, SADB_S_, γ-*TUBULIN* shRNA, WT-Nγ-tubGFP (γ-tubulin(1-333)), Ser^385^-Cγ-tubGFP (γ-tubulin(334-452)), His_6_-γ-tubulin, His_6_-Ala^131^-γ-tubulin, and GST-γ-tubulin were prepared as reported previously (2, 8). All various recombinant GFP-tagged γ-tubulin proteins were C-terminally tagged with GFP. hSadB_S_ was amplified from human cDNA by PCR, was subcloned in-frame into pGEX2T (GE Healthcare) or into the mammalian expression vector pcDNA3.1 (Invitrogen) using the following primers: 5′-CGCGGATCCACCATGTCGTCCGGGGCCAAGGA-3′ and 5′-CGCGAATTCCCTCCTCACTGCGCAGCTC-3′; 5′-GCGAAGCTTACCATGGATTATAAAGATGATGATGATAAAATGTCGTCCGGGGCCAAGGA-3′and 5′-CGCGAATTCTTACTCCTCACTGCGCAGCT-3′.Human γ-tubulin fragments and His_6_-Asp^131^-γ-tubulin were obtained by PCR from γ-tubulin/pcDNA3-GFP and Asp^131^-γ-tubulin/pcDNA3-GFP (2), respectively, and cloned into pET21d (Novagen) using the following primers: 5′-GCGGAATTCGTAACCCATCCTTCTCC-3′ and 5′-CGCAAGCTTGACCTGGGTGGGGT-3′ (human γ-tubulin(222-334)); 5′-GCGGAATTCGTCACAAGAGCTTGCAG-3′ and 5′-GCGAAGCTTCTGGGTGCCCCAGGA-3′ (P1) (human γ-tubulin(335-451)); and GCGGAATTCGTATGCCGAGGGAAATCATCACC and P1 (human Asp^131^-γ-tubulin). Ser^385^ and Ser^383^ were replaced in the various constructs using a QuikChange site-directed mutagenesis kit (Stratagene) and the following primers (mutated bases underlined): 5′-GATGGCCAACCACACCAGCATCGATTCGCTCTTCGAGAGAACCTGTCG-3′ and 5′-CGACAGGTTCTCTCGAAGAGCGAATCGATGCTGGTGTGGTTGGCCATC3′ (S385D); 5′-GGCCAACCACACCAGCATC*G*CCTCGCTCTTCGAGAGAAC-3′ and 5′-GTTCTCTCGAAGAGCGAGG*C*GATGCTGGTGTGGTTGGCC-3′ (S385A); and 5′-CATGATGGCCAACCACACC*G*GCATCTCCTCGCTCTTCG-3′ and 5′-GCAAGAGCGAGGAGATGCCGGTGTGGTTGGCCATCATG-3′ (S383G). Each mutation was verified by sequence analysis.

The following antibodies and reagents were used: anti-GCP2 (Atlas Antibodies); anti-histone (Chemicon/Millipore); anti-GCP3 anti-GFP, anti-cyclin E, anti-cyclin A, anti-E2F1, and anti-cyclin B (Santa Cruz Biotechnology, Inc.); anti-γ-tubulin (T 3320 rabbit polyclonal anti-C-terminal γ-tubulin and T 6557 mouse monoclonal anti-N-terminal γ-tubulin) and anti-FLAG (Sigma); anti-α-tubulin (Calbiochem); anti-cyclin D (Cell Signaling); anti-phospho-histone H1 (Upstate Biotechnology, Inc.); protein G PLUS-agarose, protein A PLUS-Sepharose, and [γ-^32^P]ATP (GE Healthcare); and SDS-PAGE reagents (Bio-Rad). All other reagents were obtained from Sigma.

##### Cell Culture, Fractionation, Transfection, and Cell Cycle Analysis

NIH3T3 cells and U2OS cells were cultured, transfected, and fractionated as described (2, 8, 21). In brief, fractionated cells were lysed in buffer containing 0.1% Triton X-100 (BADT), and the supernatant was the cytosolic fraction. Thereafter, the supernatant of lysed nuclei was the nuclear membrane fraction, and the remaining pellet was the chromatin fraction (21). The amount of microtubule components attached to the nuclear membrane varies a lot between experiments (8). The purified fractions were analyzed by Western blotting using α-tubulin and histone as molecular markers for the cytosolic and nuclear fractions, respectively (8). Alternatively, the different fractions were resuspended in 1× gtub buffer (50 mm Tris, (pH 7.5), 150 mm NaCl, 1 mm dithiothreitol, 1 mm EGTA, 1 mm MgCl_2_, 0.1 mm GTP, 0.5% Triton X-100, 0.1 mm Na_3_VO_4_, 2 μg/ml aprotinin, 10 μm leupeptin, 1 μg/ml pepstatin, and 1 mm phenylmethylsulfonyl fluoride) and immunoprecipitated, as described (2, 8). For cell synchronization, serum-arrested NIH3T3 cells were released for different time periods, as described previously (2, 8). To arrest cells at early S phase, U2OS cells were presynchronized by treatment with 2 mm thymidine for 16 h and then washed and incubated in normal growth medium for 8 h, followed by a second 2 mm thymidine treatment for an additional 16 h. Then cells were harvested (0 h) or washed and incubated in normal growth medium for various times (22). Otherwise, to arrest cells in G1, U2OS cells were kept at confluence during 48 h. Cell cycle progression was analyzed by flow cytometry (2).

To obtain more equal protein levels of the various γ-tubGFP mutants, the following DNA amounts were used in transfection experiments: 2 μg of Ser^385^-Cγ-tubGFP, 1 μg of Ala^385^-Cγ-tubGFP, 3 μg of Asp^385^-Cγ-tubGFP, or 1 μg of GFP. In addition, U2OS cells were simultaneously transfected and thymidine-presynchronized as mentioned above.

##### Microscopy

NIH3T3 or U2OS cells were cultured and fixed as reported (2). Cells were incubated (1 h) with primary antibody, washed, and incubated (1 h) with Alexa488- or Cy3-labeled secondary antibody (Jackson), as described (2). Images were captured using an Olympus Bx51 or an Olympus IX73 microscope. Nearly simultaneous confocal GFP/differential interference contrast imaging sequences were collected using a Zeiss Axio Observer microscope (×63, 1.4 numerical aperture plan-apochromat lens) with a stepper motor control for *z*-positioning and an Upgrade kit Axio Observer camera. Time lapse images were captured every 2 min. Time intervals of the mitotic processes were determined by counting film frames. Images were processed using ImageJ software. A minimum of 100 cells were counted in each sample, and the percentage of cells containing higher phospho-Ser^385^ (Ser(P)^385^)-γ-tubulin levels in the centrosomes or nucleus was calculated.

##### RACE-PCR Analysis

Total RNA was isolated from U2OS cells as described elsewhere (2). For RACE-PCR, first-strand cDNA was synthesized using a Smart RACE kit (Clontech) and total RNA. The following targets were used to identify the N and C termini of SadB_S_: 5′-TCGGGCCGGGACCAAGGGCACCATGT-3′ and 5′-TCGGGCCTCCTTGGGCGTCAGTCTCCC-3′ (N terminus); 5′-CCTGGTTCTGGAGCACGTCTCGGG-3′ and 5′-CGGGTGCAGGGGTCTTGGGGTCTTACTC-3′ (C terminus). The entire SadB_S_ sequence was then cloned into the HindIII/EcoRI sites of pcDNA3 (Invitrogen) with the FLAG epitope introduced 5′ of hSadB_S_ or into BamHI/EcoRI sites of pGEX2T. We encountered difficulties in cloning *SADB*_S_, because *Escherichia coli* DH5α frequently deleted the kinase domain, thereby causing a frameshift mutation creating a nonsense codon. One of the recovered mutants was SadB_S_^Δ61–198^, which was cloned into pET21d using the primers 5′-CGCGAATTCACCATGTCGTCCGGGGCCAA-3′ and 5′-GCGAAGCTTCTCCTCACTGCGCAGCTC-3′. The gene carried a nonsense codon that was removed using the QuikChange site-directed mutagenesis kit and these primers (inserted bases underlined): 5′-CCCATTATGCGTGGCTCCAGAGGTGATTAAG-3′ and 5′-CTTAATCACCTCTGGAGCCACGCATAATGGG-3′.

##### Gene Expression Analysis and Luciferase Assays

Total RNA isolation was performed as described previously (8). mRNA expression array analysis was performed using the human Illumina platform. Data were normalized using quantile normalization, and the analysis of differential expression was performed using a linear model fitting (LIMMA packages) as described previously (23). Heat maps representing the expression intensity were drawn using the R function heatmap.2 in the gplots package (G. R. Warnes, B. Bolker, and T. Lumley, gplots:Various R programming tools for plotting data, R package version 2.6.0). Unsupervised clustering was performed using the R function hclust (method = “ward”). Luciferase assays were performed on transfected U2OS cells, as described elsewhere (8).

##### Antibody Production, Immunoprecipitation, and SADB Kinase Assay

A rabbit anti-Ser(P)^385^-γ-tubulin antibody was generated using the phosphopeptide RVSGLMMANHTSISSLFE (phosphorylated Ser underlined; Pacific Immunology) and was purified as described (2).

Total lysates from cells, SadB kinases, and HA- and FLAG-tagged immunoprecipitates were prepared as reported (2, 8). To increase the affinity of the rabbit polyclonal anti-SADB antibody (2) in Western blot analysis, we mixed it 5:1 with rabbit polyclonal anti-N-terminal SadB (Abcam) antibody.

The SADB kinase assay and λ-phosphatase treatment were conducted as described (2). The N-terminal GST SadB_S_ was expressed in DH5α, and exponentially growing bacteria were induced with 0.2 mm isopropyl-1-thio-β-d-galactopyranoside at room temperature overnight. GST-γ-tubulin, C-terminal His_6_-tagged human SadB_S_^Δ61–198^, WT-γ-tubulin, Ala^131^-γ-tubulin, Asp^131^-γ-tubulin, WT-γ-tubulin(222-334), WT-γ-tubulin(335-451), Gly-383-γ-tubulin(335-451), or Ala^385^-γ-tubulin(335-451) was purified as described (2). SadB_S_ and γ-tubulin were excised from GST using thrombin (Amersham Biosciences).

##### Statistical Analysis

All data are expressed as mean ± S.D. (*n* < 4) or S.E. (*n* ≥ 4), and Student's paired *t* test was used to analyze differences. Cell cycle profiles were assessed using FlowJo (Tree Star, Inc.). Western blotting bands were quantitated with ImageJ software.

## RESULTS

### 

#### 

##### Two Biochemically Different Pools of γ-Tubulin in Mammalian Cell Lines

**FIGURE 1.**
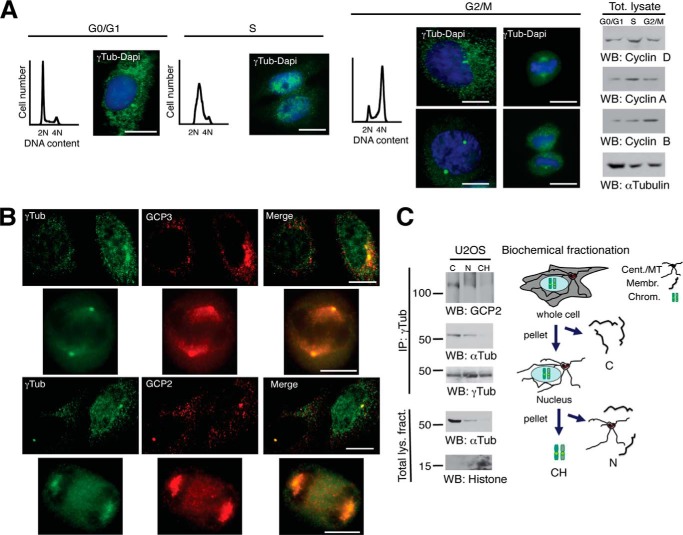
**Nuclear γ-tubulin is not associated with GCP2 or GCP3.** *A*, cells were synchronized in G_0_/G_1_ by keeping cell confluence during 48 h (G_0_/G_1_) or in early S phase by double thymidine block and released for 5 h (S) or 9 h (G_2_/M). Cell cycle progression was monitored by determining the DNA content of cells by flow cytometry (*graphs*) and by analyzing the protein levels of the G_1_ marker cyclin D, the S-phase marker cyclin A, and the G_2_/M marker cyclin B in cell lysates (*Tot. lysate*) by Western blotting (*right panels*). *A* and *B*, localization of endogenous γ-tubulin was examined by immunofluorescence staining with anti-γ-tubulin (*green*; rabbit) and anti-GCP3 (*red*; mouse) or anti-γ-tubulin (*green*; mouse) and anti-GCP2 (*red*; rabbit) antibodies, and nuclei were detected using DAPI (*blue*) in U2OS cells (*n* = 3–5). *Scale bars*, 10 μm. *C*, cells (20 × 10^6^) were biochemically divided into the following cell fractions: cytosolic (*C*), nuclear membrane (*N*), and chromatin (*CH*). Each fraction was subjected to immunoprecipitations (*IP*) with an anti-γ-tubulin antibody and developed by Western blotting (*WB*) with an anti-GCP2 antibody (*top*), and then reprobed with α-tubulin (α*Tub*) and γ-tubulin (γ*Tub*). Aliquots of the lysates used in the immunoprecipitations were run as loading controls (*Total lys. fract*.) and analyzed by Western blotting (*n* = 3). Models depict the cellular distribution of centrosomes/microtubules (*Cent*/*MT*), membranes (*Membr*.), and chromosomal (*Chrom*.) elements in the analyzed biochemical fractions.

To visualize the cellular localization of γ-tubulin during cell cycle (8, 10, 11), we performed immunofluorescence analysis with previously characterized antibodies (8, 9) of a synchronized cell population (Fig. 1, *A* and *B*). Upon S phase entry, γ-tubulin accumulated in the nucleus (8, 10, 11) and remained nuclear throughout S phase (Fig. 1*A*). During G_2_ phase, a successive decrease of chromatin-bound γ-tubulin was observed, reaching the lowest level in mitotic chromosomes (Fig. 1*A*). Finally, at early mitosis, γ-tubulin was found in the cytosol and centrosomes (Fig. 1, *A* and *B*). In most eukaryotic cells, γ-tubulin links a growing microtubule to a γTuRC by interacting with GCP2 and GCP3 (4). To study a possible association of other γTuRC components to chromatin, we analyzed the localization of GCP2 and GCP3 by immunostaining (Fig. 1*B*) and of GCP2 and α-tubulin by Western blotting (Fig. 1*C*). Although the localization of γ-tubulin varied (Fig. 1, *A* and *B*), GCP2 and GCP3 occurred only in the cytosol, centrosomes, and mitotic spindles (Fig. 1*B*). Analysis of biochemical fractionations (21) confirmed that GCP2 and α-tubulin only associated with γ-tubulin immunoprecipitates from cytosolic and nuclear membrane fractions (Fig. 1*C*). These results indicate that the composition of chromatin-associated γ-tubulin complexes differs from γTuRC.

##### Increased Expression of SadB Augments the Size of the Nuclear γ-Tubulin Pool

**FIGURE 2.**
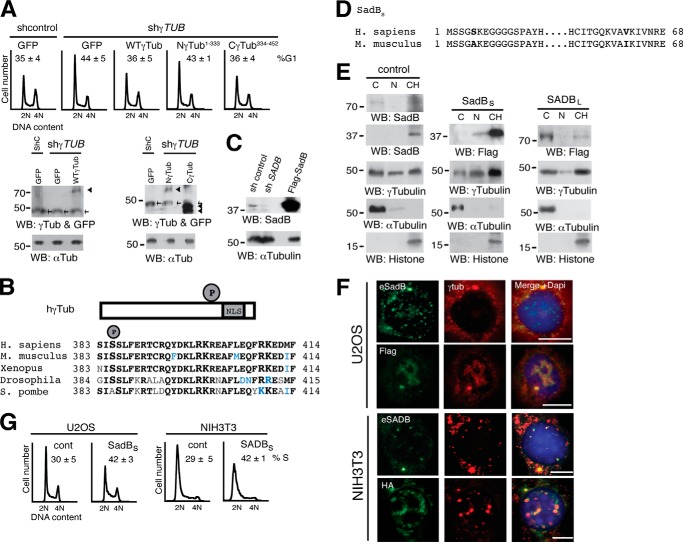
**An increased level of SadB_S_ augments the nuclear pool of γ-tubulin.** *A*, the DNA content was determined by flow cytometry of non-synchronous U2OS cells expressing shRNA (*shcontrol*; *shC*) or sh-γ-*TUBULIN* (*sh*γ*TUB*) and co-transfected with one or the following constructs: GFP, wild-type γ-tubGFP (*WT-*γ-*Tub*), Nγ-tubGFP (*N*γ-*Tub^1–333^*), or Cγ-tubGFP (*C*γ-*Tub^334–452^*), as indicated. The data on each cell population are presented as the proportion of cells in G_1_ (*n* = 3). *Bottom*, cell extracts were analyzed by Western blotting. *Arrowheads* and *arrows* show GFP and endogenous γ-tubulin, respectively (*n* = 3). *B*, structure of wild-type human γ-tubulin (*h*γ*Tub*) constructs depicting the NLS and phosphorylated Ser^385^ (*P*). The region surrounding Ser^385^ is conserved in the indicated species. *Boldface letters*, identity; *blue letters*, polar or hydrophobic conservation. *C*, total lysate of U2OS cells transfected with a control vector, *SADB* shRNA, or FLAG-SadB_S_ and analyzed by Western blotting (*WB*) using an anti-SadB antibody, followed by anti-α-tubulin (*n* = 3). *D*, structure of human SadB_S_ (*top*) and mouse SADB_S_ (*bottom*) constructs showing the amino acids that differ between the two isoforms in *boldface type. E*, U2OS cells (1 × 10^6^) expressing control, FLAG-tagged human SadB_S_, or mouse SADB_L_ vectors were biochemically divided into cytosolic (*C*), nuclear membrane (*N*), and chromatin (*CH*) fractions, as in Fig. 1*C*, and analyzed by Western blotting (*WB*; *n* = 5). *F*, localization of endogenous human and mouse SadB (*eSadB* and *eSADB*, respectively), FLAG-SadB_S_, HA-SADB_S_, and endogenous γ-tubulin was examined by immunofluorescence staining with SADB_S_, FLAG, or HA (*green*) and γ-tubulin (*red*). Nuclei were detected using DAPI (*blue*) in transfected human U2OS and mouse NIH3T3 cells (*n* = 5). Fluorescence intensity of endogenous nuclear γ-tubulin staining in cells expressing FLAG-SadB_S_ or HA-SADB_S_ was quantified relative to control cells. *Scale bars*, 10 μm. *G*, flow cytometry was performed to determine DNA content in control NIH3T3 or U2OS cells transfected with FLAG-SadB_S_ (*SadB_S_*) or HA-SADB_S_ (*SADB_S_*), as indicated. The percentage of S phase cells is shown in each *panel* (*n* = 3).

To identify the domain in γ-tubulin important for its effect on S-phase entry (8), we tested the effect of various γ-tubulin mutants tagged with green fluorescence protein (GFP; γ-tubGFP) on the cell cycle. This revealed that U2OS cells transfected with γ-*TUBULIN* shRNA accumulated in G_1_ phase (*n* = 3, *p* < 0.05), an effect that was rescued by ectopic expression of either an sh-resistant γ-*TUBULIN* gene (*n* = 3, *p* < 0.05) or an sh-resistant C γ-tubulin terminus (Cγ-tubGFP; *n* = 3, *p* < 0.05) but not by expression of the sh-resistant γ-tubulin N terminus (Nγ-tubGFP) (8), suggesting that the γ-tubulin domain that determines optimal cell cycle progression is the C-terminal region of γ-tubulin (Fig. 2*A*).

We have previously shown that the C terminus of γ-tubulin (γ-tubulin(334-452)) encompasses the DNA-binding domain and the NLS, the latter of which includes residues Arg^399^, Lys^400^, Arg^409^, and Lys^410^ (Fig. 2*B*) (1, 8). Phosphorylation of residues near an NLS is often done to induce a conformational change to unmask the NLS and thereby trigger translocation of a cytosolic protein to the nucleus (24). Applying that strategy, we found the SadB phosphorylation motif Ser-*X*-Ser (Ser^383^-Ile^384^-Ser^385^) 14 amino acids upstream of Arg^399^ (Fig. 2*B*) (8). To investigate whether hSadB affects the nuclear γ-tubulin pool, we cloned h*SADB_Short_* (*SADB_S_*) (GenBank^TM^ accession number HQ830199) from U2OS cells. Expression of the *SADB* gene product, SadB_S_, was decreased in U2OS cells expressing *SADB* shRNA (Fig. 2*C*) (2). Human SadB_S_ and mouse SADB_S_ (2) protein sequences showed 99% homology, with only two amino acids differing: Ser^5^ and Val^62^ in the former but Ala^5^ and Ile^62^ in the latter (Fig. 2*D*). We have previously demonstrated that SADB kinases localized to centrosomes, but the exact cellular localization of the various SADB isoforms has not yet been elucidated. Biochemical fractionations of U2OS cells showed disparate localization of SadB_L_ and SadB_S_. Endogenous SadB_L_ and recombinant FLAG-SADB_L_ localized to cytosolic and chromatin fractions (15), whereas endogenous SadB_S_ and recombinant FLAG-SadB_S_ were found mainly in chromatin fractions (Fig. 2*E*). Notably, elevated expression of mouse SADB_L_ or hSadB_S_ increased the pool of chromatin-bound γ-tubulin (Fig. 2*E*). A densitometric analysis of Western blots containing the different cellular fractions showed that increase protein levels of SadB_S_ caused a 67.2% (29.3% ± 4.4 control and 49.0% ± 1.7 SadB_S_; *n* = 3) rise of the amount of endogenous γ-tubulin in the chromatin (Fig. 2*E*). Immunofluorescence analysis of endogenous SadB and FLAG-SadB_S_ and HA-SADB_S_ confirmed that SadB_S_ localized not only to centrosomes (1) but also to the nucleus (Fig. 2*F*). Furthermore, compared with control cells, increased expression of SadB_S_ raised the nuclear γ-tubulin content by 84% in U2OS (*n* = 8) and with 72% (*n* = 8) in NIH3T3 cells, creating distinct γ-tubulin domains in the nuclei of these cells (Fig. 2*F*). Thus, augmented nuclear γ-tubulin levels coincided with an accumulation of cells in S phase (Fig. 2*G*; *n* = 3, *p* < 0.01) (4). These findings suggest a role for SadB_S_ in regulation of the nuclear localization of γ-tubulin.

##### SadB Levels Affect Phosphorylation of Ser^385^ in γ-Tubulin and Cell Cycle Progression

**FIGURE 3.**
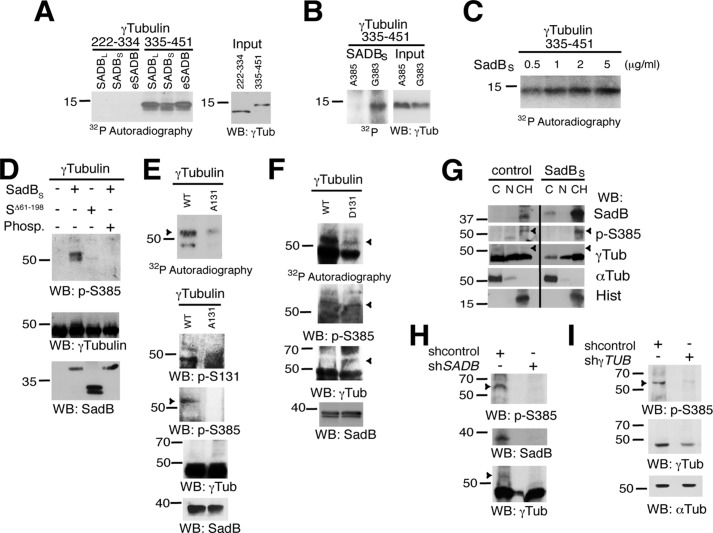
**SadB kinases phosphorylate γ-tubulin on Ser^385^ and modulate Ser(P)^385^-γ-tubulin cellular levels.** *A* and *B*, fractions of FLAG-SADB_S_-, HA-SADB_S_-, or control-expressing NIH3T3 cells were immunoprecipitated using anti-FLAG, anti-HA, or anti-SADB antibodies (*eSADB*). Parallel samples of immunopurified kinases were tested in *in vitro* kinase assays using bacterially produced His_6_-γ-Tub(222–334), His_6_-γ-Tub(335–451), Ala^385^-γ-Tub(335–451), and Gly^383^-γ-Tub(335–451) fragments, as indicated (*n* = 3). *C–F*, bacterially produced SadB_S_ and γ-tubulin was excised from GST using thrombin. *C*, the figure shows the activities exhibited of various concentrations of SadB_S_ using the His_6_-γ-Tub(335–451) fragments (*n* = 2). *D*, kinase assays were performed as in *A* using non-radioactive ATP in the presence or absence of λ-phosphatase (*n* = 3). The figure shows SadB_S_ or SadB_S_^Δ61–198^ (*S*^Δ61–198^) activities exhibited using full-length human His_6_-γ-tubulin. *E* and *F*, the ability to phosphorylate Ser^385^-γ-tubulin of bacterially produced SadB_S_ was tested *in vitro* by using radioactive or non-radioactive ATP and bacterially produced His_6_-γ-tubulin, His_6_-Ala^131^-γ-tubulin (*A131*), or His_6_-Ala^131^-γ-tubulin (*D131*) and SadB_S_, as indicated. The non-radioactively labeled samples were analyzed by Western blotting (*WB*). Ser(P)^385^-γ-tubulin and Ser^131^-γ-tubulin levels were examined by using first an anti-Ser(P)^385^-γ-tubulin (*p-S385*) antibody and thereafter using anti-Ser(P)^131^-γ-tubulin (*p-S131*), anti-γ-tubulin (γ*Tub*), and anti-SadB, as indicated. *Arrowheads*, Ser(P)^385^-γ-tubulin (*n* = 3). *G–I*, U2OS cells were transfected with a control shRNA (*shcontrol*), FLAG-SadB_S_, *SADB* shRNA- (*shSADB*), or γ*TUBULIN* shRNA (*sh*γ*TUB*), and cell lysates were analyzed by Western blotting as in Fig. 2, *C* and *E* (*n* = 3). Endogenous phosphorylated Ser^385^-γ-tubulin (*pSer-385-*γ*-tubulin*) levels were examined by using first an anti-Ser(P)^385^-γ-tubulin (*p-S385*) antibody and thereafter using anti-SadB, anti-γ-tubulin (γ*Tub*), anti-α-tubulin (α*Tub*), and anti-histone (*Hist*), as indicated. *Arrowheads*, Ser(P)^385^-γ-tubulin (*n* = 3).

To detect whether SADB phosphorylates Ser^385^ in the motif Ser^383^-Ile^384^-Ser^385^ (Fig. 2*B*), we investigated SADB molecules in kinase assays using various recombinant proteins as substrates. Immunopurified endogenous SADB and recombinant FLAG-SADB_L_ and HA-SADB_S_ phosphorylated bacterially produced human γ-tubulin(335-451) but not the corresponding fragment γ-tubulin(222-334), which lacks Ser^385^ (Fig. 3*A*). Moreover, SADB_S_ phosphorylated the Gly^383^-γ-tubulin(335-451) but not the Ala^385^-γ-tubulin(335-451) substitution mutant (Fig. 3*B*). Finally, bacterially produced hSadB_S_ phosphorylated the γ-tubulin(335-451) fragment (Fig. 3*C*). We noticed that the motif surrounding Ser^385^ is conserved in vertebrates and most invertebrates but not in the fission yeast *Schizosaccharomyces pombe*, which has an Ala instead of Ser^385^ but nonetheless highly conserved Ser^383^, Ser^386^, and motifs surrounding Ala^385^ (Fig. 2*B*). These findings suggest that the functions of this region in γ-tubulin are conserved among species.

To examine the endogenous Ser(P)^385^-γ-tubulin levels and their potential dependence on SadB in cells, we prepared an anti-Ser(P)^385^ antibody that recognized purified bacterially produced full-length human γ-tubulin that had been phosphorylated in *in vitro* by purified bacterially produced SadB_S_, and notably, this signal was reduced by phosphatase treatment or incubation with the kinase-dead mutant SadB_S_^Δ61–198^ (Fig. 3*D*). To study a possible link between phosphorylation of Ser^131^-γ-tubulin and of Ser^385^, we analyzed the effect of phosphorylation on Ser^385^ in a non-phosphorylatable Ser^131^ → Ala γ-tubulin (Fig. 3*E*) and a phosphomimetic Ser^131^ → Asp γ-tubulin (Fig. 3*F*). We found that phosphorylation of Ser^385^ was reduced in the Ala^131^-γ-tubulin mutant but restored in the Asp^131^-γ-tubulin mutant, suggesting that phosphorylation of Ser^131^ is a prerequisite for the phosphorylation of Ser^385^. However, the band detected by the anti-Ser(P)^385^ antibody had a higher molecular mass than expected (60,000 Da) and was not detected by total anti-γ-tubulin antibody (Fig. 3, *D–F*) but was recognized by anti-Ser(P)^131^ antibody (Fig. 3*E*) (2). Altogether, the results indicate that *in vitro* SadB kinases phosphorylate γ-tubulin on Ser^385^ and on Ser^131^.

To determine whether the expression levels of SadB or γ-tubulin affected the phosphorylation levels of Ser^385^-γ-tubulin, we varied their protein levels in U2OS cells. Analysis of U2OS cellular extracts using the anti-Ser(P)^385^ antibody showed an ∼60,000 Da band, which levels were affected upon increased recombinant SadB_S_ levels (Fig. 3*G*) or reduced endogenous SadB (Fig. 3*H*) or γ-tubulin expression (Fig. 3*I*). These data show that the 60 kDa band is γ-tubulin and that SadB_S_ regulates the cellular levels of Ser(P)^385^-γ-tubulin.

**FIGURE 4.**
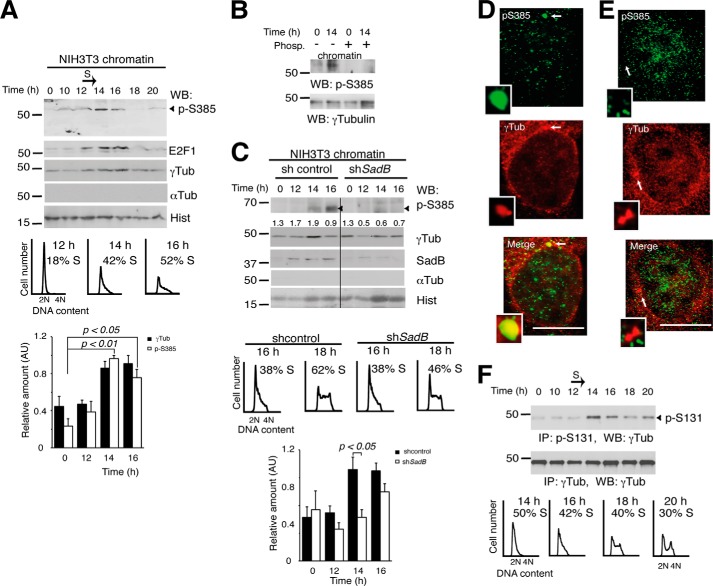
**Cellular levels of Ser(P)^385^-γ-tubulin fluctuate during cell cycle and are SadB-dependent.** *A–C*, cells were synchronized in G_0_ and released for different time periods. The samples were biochemically fractionated, and the chromatin fractions were analyzed as in Fig. 2*E*. The DNA content of the synchronous NIH3T3 cells was determined by flow cytometry. The percentage of S phase cells is indicated in each *panel* (*n* = 4). *A*, *bottom*, graph shows the mean γ-tubulin (γ*Tub*) and Ser(P)^385^-γ-tubulin signal found in chromatin fractions expressed in arbitrary units (AU; mean ± S.E. (*error bars*); *n* = 5). *B*, chromatin extracts of synchronous NIH3T3 cells were or were not treated with λ-phosphatase (*Phosp*.). *C*, control shRNA-transfected (*shcontrol*) or SadB shRNA-transfected (*shSadB*) NIH3T3 cells were analyzed as in *A* (*n* = 3). *Numbers* on the Western blot indicate variations on γ-tubulin expression relative to control. To adjust for differences in protein loading, the protein concentration of γ-tubulin was determined by its ratio with histone for each treatment. *Bottom*, graph illustrates the mean value of the Ser(P)^385^-γ-tubulin signal in chromatin fractions from control shRNA- or SadB shRNA-transfected NIH3T3 cells (mean ± S.E.; *n* = 5). *D* and *E*, localization of endogenous Ser(P)^385^-γ-tubulin was examined by immunofluorescence staining with anti-Ser(P)^385^-γ-tubulin (*green*; rabbit) and anti-γ-tubulin (*red*; mouse), and nuclei were detected using DAPI (*blue*) in human S phase-synchronized U2OS cells that were released for 5 h. *D*, a U2OS cell containing higher Ser(P)^385^-γ-tubulin levels in the centrosomes. *E*, a U2OS cell containing higher nuclear levels of Ser(P)^385^-γ-tubulin and nuclear γ-tubulin. *Arrows* show the location of centrosomes (*n* = 3). *Inset*, higher magnification. *Scale bars*, 10 μm. *F*, *top*, Ser(P)^131^-γ-tubulin cell content of a cell population treated as in *A* was examined by immunoprecipitation (*IP*) of Ser(P)^131^-γ-tubulin from synchronous NIH3T3 cell extracts, followed by Western blot (*WB*) with γ-tubulin Ab. Total γ-tubulin levels were examined in cell extracts by immunoprecipitation with anti-γ-tubulin (*n* = 3). *Bottom*, flow cytometry was performed to determine DNA content in NIH3T3 cells. The percentage of S phase cells is shown in each *panel* (*n* = 3).

We subsequently examined endogenous Ser(P)^385^-γ-tubulin levels in synchronized NIH3T3 cells. Western blotting of chromatin fractions of NIH3T3 cells with Ser(P)^385^ antibody revealed accumulation of endogenous Ser(P)^385^-γ-tubulin during early S phase (Fig. 4*A*). The 60 kDa band recognized by anti-Ser(P)^385^ antibody was phosphatase-sensitive (Fig. 4*B*), and increased Ser(P)^385^-γ-tubulin levels coincided with a rise in nuclear γ-tubulin and E2F1 proteins (Fig. 4*A*). In contrast, decreased SADB levels reduced the transient increase in nuclear γ-tubulin and Ser(P)^385^-γ-tubulin and also delayed S-phase entry (Fig. 4*C*) (2). Finally, immunofluorescence analysis of S-phase-synchronized U2OS cells (Fig. 1*A*) showed that Ser(P)^385^-γ-tubulin localized to centrosomes (Fig. 4*D*; 10 ± 2%; *n* = 3) and chromatin (Fig. 4*E*; 23 ± 3%; *n* = 3). Together, our data indicate that the transient increase in nuclear γ-tubulin during early S phase is caused by SadB-mediated phosphorylation of Ser^385^.

During cell division, the activity of SadB follows the replication of centrosomes and chromosomes by phosphorylation of γ-tubulin on Ser^131^ (2). In this way, SadB enhances γ-tubulin polymerization at the nascent centriole and inhibits acentriolar formation of centrosomes elsewhere in the cell (2, 16). To further elucidate a possible interdependence between the phosphorylation pattern of Ser^131^ and Ser^385^, we examined the endogenous Ser(P)^131^-γ-tubulin levels in synchronized NIH3T3 cells (Fig. 4*F*). Western blot analysis of Ser(P)^131^ immunoprecipitates (2) revealed high levels of Ser(P)^131^ in early S and G_2_/M phases (Fig. 4*F*), a pattern that coincided with the replication of centrosomes and the formation of the mitotic spindle (2, 8, 12), suggesting that phosphorylation on Ser^385^ and on Ser^131^ regulates different cellular processes. However, although the phosphorylation levels differ, both phosphorylations were detectable at 14 h (Fig. 4, *A* and *F*), implying a possible interrelationship. In support of this view, *in vitro* phosphorylation in Ser^385^ was altered in an Ala^131^-γ-tubulin and Asp^131^-γ-tubulin mutants (Fig. 3, *E* and *F*), and endogenous Ser(P)^385^-γ-tubulin had a centrosomal localization (Fig. 4*D*). Based on our results, we postulate that phosphorylation of Ser^131^ (1, 6) liberates γTURCs from αβ-tubulin dimers (2) and in this way allows the transient phosphorylation of Ser^385^-γ-tubulin (Fig. 4, *A* and *C*).

##### Phosphorylation Levels of Ser^385^-γ-Tubulin Control the Size of the Nuclear γ-Tubulin Pool and Gene Expression

**FIGURE 5.**
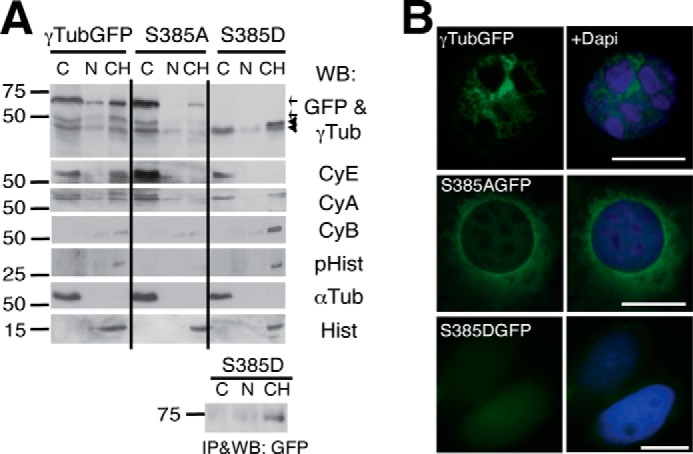
**Ser^385^-γ-tubGFP mutants affect the nuclear localization of γ-tubulin.** *A* and *B*, U2OS cells transfected with γ-tubGFP, Ala^385^-γ-tubGFP (S385A), or Asp^385^-γ-tubGFP (S385D) were examined by Western blotting (*A*) and immunofluorescence microscopy (*B*). *A*, the various biochemical fractions obtained from U2OS cells were analyzed by Western blotting (*WB*) with anti-GFP (*GFP*), anti-γ-tubulin (γ*Tub*), anti-cyclin E (*CyE*), anti-cyclin A (*CyA*), anti-cyclin B (*CyB*), anti-phospho-histone H1 (*pHist*), anti-α-tubulin (α*Tub*), and anti-histone (*Hist*) antibodies (*n* = 3) as in Fig. 2*E*. Anti-GFP antibody immunoprecipitates of cell lysates prepared as described in Fig. 2*E* but expressing Asp^385^-γ-tubGFP were analyzed by WB. *Arrows* and *arrowheads* indicate the GFP and endogenous γ-tubulin, respectively. *B*, the cellular location of γ-tubGFP, Ala^385^-γ-tubGFP (*S385AGFP*), and Asp^385^-γ-tubGFP (*S385DGFP*) was determined by immunofluorescence analysis, and nuclei were detected using DAPI (*blue*); *scale bars*, 10 μm.

To determine whether Ser^385^-γ-tubulin phosphorylation regulates the cellular location of γ-tubulin, we analyzed expression of a non-phosphorylatable Ala^385^-γ-tubulin or a phosphomimetic mutant Asp^385^-γ-tubulin tagged with green fluorescence protein (GFP; γ-tubGFP) in U2OS cells. Biochemical fractionation analysis of U2OS cells transiently expressing the non-phosphorylatable Ala^385^-γ-tubGFP mutant exhibited a reduced amount of chromatin-associated endogenous γ-tubulin and recombinant Ala^385^-γ-tubGFP (Fig. 5*A*). In contrast, ectopic expression of Asp^385^-γ-tubGFP increased the levels of endogenous nuclear γ-tubulin (γ-tubGFP, 33.8 ± 3.0%; Ala^385^-γ-tubGFP, 16.8 ± 2.5%; Asp^385^-γ-tubGFP, 47.3 ± 18.0%; *n* = 3) in the studied cell populations (Fig. 5*A*). Considering the opposite effects of Ala^385^-γ-tubGFP and Asp^385^-γ-tubGFP mutants on the accumulation of endogenous γ-tubulin in chromatin fractions, we propose that phosphorylation of Ser^385^ initiates a signal cascade that mediates translocation of phosphorylated and non-phosphorylated γ-tubulin molecules to the chromatin.

Unfortunately, expression of the Asp^385^-γ-tubGFP was not detectable by Western blotting of lysates of 1 × 10^6^ cells (Fig. 5*A*), and immunofluorescence evaluation showed a very weak nuclear signal (Fig. 5*B*). To ensure the chromatin localization of Asp^385^-γ-tubGFP, we immunoprecipitated it from fractions of 6 × 10^6^ U2OS cells (Fig. 5*A*). Asp^385^-γ-tubGFP occurred mainly in the chromatin fraction, suggesting that the phosphorylation levels of Ser^385^ modulate the nuclear function of γ-tubulin. Accordingly, immunofluorescence analysis (Fig. 5*B*) showed that γ-tubGFP was found in the nucleus of a larger number of U2OS cells (24% ± 4.4; *n* = 3) than the Ala^385^-γ-tubGFP mutant (8% ± 2.3; *n* = 3).

**FIGURE 6.**
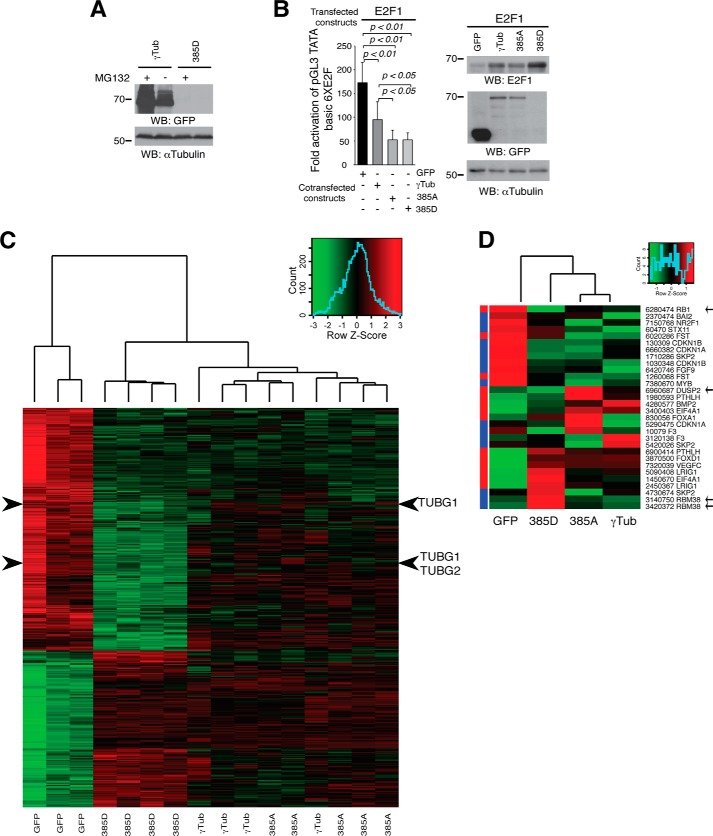
**Ser^385^-γ-tubGFP mutants moderate transcriptional activity during cell cycle.** *A*, U2OS cells expressing γ-tubGFP (γ*Tub*) or Asp^385^-γ-tubGFP (*D385*γ*Tub*) were treated overnight in the presence or absence of 10 μm MG132. The indicated antibodies were used to analyze Western blot (*WB*) of total cell lysates (*n* = *2*). *B*, assay of the luciferase activity driven by six E2F promoter binding sites on transient transfection of U2OS cells with a *Renilla* reporter construct and the following constructs: HA-E2F1, GFP, γ-tubGFP (γ*Tub*), Ala^385^-γ-tubGFP (385A), or Asp^385^-γ-tubGFP (*385D*), as indicated, is shown at the *left*. Luciferase activity of cells transfected with control construct was set as 1, and relative activities were calculated (mean ± S.D.; *n* = *3*). At the *right* are shown total lysates of transfected U2OS cells that were analyzed by Western blot with the indicated antibodies. *C*, an mRNA expression array was performed on U2OS cells ectopically expressing GFP (*n* = *3*), Asp^385^-γ-tubGFP (*385D*; *n* = *4*), γ-tubGFP (γ*Tub*; *n* = *4*), or Ala^385^-γ-tubGFP (*385A*; *n* = *5*). Comparison of gene expression profiles at the probe set level between cells ectopically expressing Asp^385^-γ-tubGFP (*385D*; *n* = 4) or GFP show that 413 genes were differentially expressed (*p* < 1 × 10^−3^). The gene expression of the 413 genes is represented in a heat map, in which *red* and *green* indicate high and low expression, respectively, according to the scale shown (*top right graph*). The *arrowheads* show the expression of TUBG1 and TUBG2. *D*, the heat map visualizes the comparative gene expression analyses at the probe set level between U2OS cells ectopically expressing GFP (*n* = *3*), Ala^385^-γ-tubGFP (*385A*; *n* = *5*), γ-tubGFP (γTub; *n* = *4*), or Asp^385^-γ-tubGFP (*385D*; *n* = *4*) of the 20 most differently expressed E2F-regulated genes upon decreased expression levels of γ-tubulin (8), in which *green* indicates low expression and *red* indicates high expression according to the *scale* shown (*top right graph*). The *left column* represents the previously reported expression changes (*red*, increased; *blue*, decreased) of the 20 E2F-regulated genes affected by decreased γ-tubulin expression (8). The *arrows* show *RB1*, *DUSP2*, and *RBM38*.

To identify the underlying cause of the low expression levels of Asp^385^-γ-tubGFP, we analyzed the effect of a proteosomal inhibitor, MG132, on the expression of the mutant protein. In comparison with the γ-tubGFP expression, the expression levels of Asp^385^-γ-tubGFP were not affected by MG132 (Fig. 6*A*), implying that premature proteosomal degradation is not the cause of the low expression. To further elucidate the reason of the low protein expression of Asp^385^-γ-tubGFP, we evaluated the effect of the various γ-tubGFP mutants on the activity of the nuclear γ-tubulin downstream target E2F1 by performing an assay using luciferase reporter plasmids containing E2F-binding sites (19). The luciferase activity measured in U2OS cells transfected with E2F1 (18) was reduced upon increased levels of γ-tubGFP or of the various Ser^385^-γ-tubGFP mutants, but both Asp^385^-γ-tubGFP and Ala^385^-γ-tubGFP exhibited a stronger moderating effect on E2F1 activity than wild-type γ-tubGFP (Fig. 6*B*). Considering these findings, we hypothesized that Ser^385^-γ-tubulin may have a regulatory impact on transcription depending on its phosphorylation status.

To identify functional difference between the various Ser^385^-γ-tubulin mutants, an mRNA expression array was performed on control GFP-transfected and the various γ-tubGFP-mutant-transfected cells, and the impact of γ-tubulin on RNA expression was examined. By comparison with control cells (GFP-transfected), expression of Asp^385^-γ-tubGFP correlated with the up-regulated expression of 162 genes, but the expression of 251 genes was down-regulated (Fig. 6*C*; *p* < 1 × 10^−3^) in an expression pattern that differed from the expression profile found in cells expressing γ-tubGFP or Ala^385^-γ-tubGFP (Fig. 6*C*). However, we identified the two γ-tubulin isoforms, *TUBG1* and *TUBG2* (25), among the Asp^385^-γ-tubGFP repressed genes, suggesting that the observed low expression levels of Asp^385^-γ-tubGFP may depend on a transcriptional feedback mechanism.

To further visualize the effect of the various mutants on known γ-tubulin downstream targets (8), the expression of the 20 most differentially regulated E2F-controlled genes upon decreased expression levels of γ-tubulin (8) were represented in a heat map (Fig. 6*D*). Indeed, the expressions of genes such as *RB1*, *DUSP2*, and *RBM38* are affected by the phosphorylation levels of Ser^385^-γ-tubulin, demonstrating that the various recombinant γ-tubulin proteins alter gene expression differently (Fig. 6, *C* and *D*).

##### Ala^385^-γ-Tubulin and Asp^385^-γ-Tubulin Affect Cell Cycle Progression

The level of chromatin-bound γ-tubulin is lowest during G_1_ and mitosis (Fig. 1, *A* and *B*) (8). Consequently, the decrease in chromatin-bound γ-tubulin upon expression of the Ala^385^-γ-tubGFP mutant may cause accumulation of cells in the G_1_ phase. Western blot analysis of U2OS cells expressing Ala^385^-γ-tubulin revealed increased cytosolic levels of the G_1_-S markers cyclin E and cyclin A, whereas there were low or undetectable amounts of the G_2_-M markers cyclin B and phosphorylated histone H1 (Fig. 5*A*). Immunofluorescence analysis showed that localization of WT-γ-tubGFP and Ala^385^-γ-tubGFP differed, being clearly nuclear for the former but mostly cytosolic for the latter. Moreover, chromatin-bound WT-γ-tubGFP accumulated in defined nuclear sites (Fig. 5*B*). Despite the low expression of Asp^385^-γ-tubGFP, the phosphomimetic mutant increased the chromatin-bound levels of endogenous γ-tubulin and of the G_2_-M markers phosphorylated histone H1 and cyclin B (Fig. 5*A*). Together, these findings support the involvement of Ser^385^-γ-tubulin in regulation of cell cycle progression (8).

**FIGURE 7.**
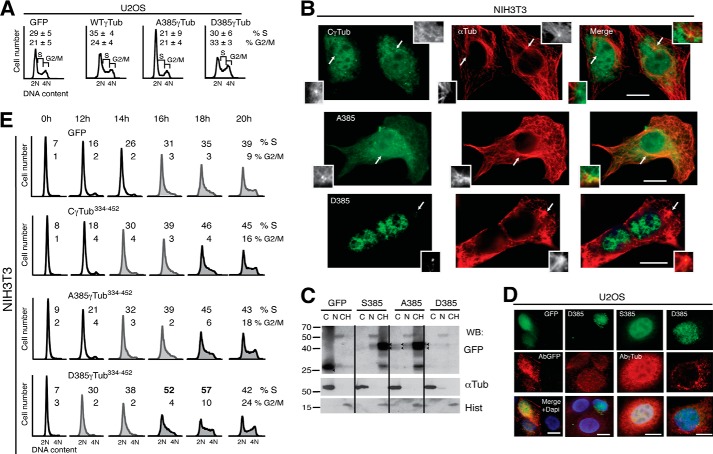
**The phosphorylation levels of Ser^385^ affect cell cycle progression.** *A* and *E*, flow cytometric analysis of the DNA content in unsynchronized U2OS or synchronized NIH3T3 cells expressing one or two of the following constructs or with the indicated C-terminal mutants: control (GFP), wild-type γ-tubGFP (*WT-*γ*Tub*), Ala^385^-γ-tubGFP (*A385*γ*Tub*), or Asp^385^-γ-tubGFP (*D385*γ*Tub*) (*A*) or Ser^385^-Cγ-tubGFP (*C*γ*Tub^334–452^*) (*E*). *A*, the data on each cell population are presented as the proportion of cells in S and G_2_/M phase (*n* = 3). *B* and *C*, localization of the indicated GFP-tagged proteins was detected in transfected NIH3T3 cells by immunofluorescence (*green*) or Western blotting with the indicated antibodies. *B*, microtubules were detected with anti-α-tubulin antibody (*red*; α*Tub*). *Arrows*, the locations of centrosomes (*n* = 3). *Inset*, higher magnification. *Scale bars*, 10 μm. *C*, cellular fractions of transfected NIH3T3 cells were analyzed as in Fig. 2*E. Arrowheads*, GFP (*n* = 2). *D*, transiently transfected U2OS cells expressing GFP (*green*), Cγ-tubGFP (*S385*; *green*), or Asp^385^-Cγ-tubGFP (*D385*; *green*) were analyzed by immunofluorescence staining with an anti-GFP (*AbGFP*; *red*) or anti-γ-tubulin (*Ab*γ-*Tub*; *red*) antibody and nuclei by DAPI staining (*blue*), as indicated. *Scale bars*, 10 μm. *E*, the data on each cell population are presented as the proportion of cells in S phase: <30% (*black open histograms*), <40% (*gray closed histograms*), and >40% (*black closed histograms*; *n* = 3).

To further characterize the involvement of Ser^385^-γ-tubulin in cell division, we examined the cell cycle profile of a population of U2OS cells expressing the various γ-tubGFP mutants (Fig. 7*A*). Compared with cells expressing GFP, those expressing WT-γ-tubGFP showed increased numbers in S phase (Fig. 7*A*) (8), similar to what was noted for cells expressing SadB_S_ (Fig. 2*G*). In contrast, expression of the Ala^385^-γ-tubGFP mutant led to accumulation of cells in G_1_ phase (Fig. 7*A*; *n* = 3, *p* < 0.05), whereas Asp^385^-γ-tubGFP expression increased the number of cells in phases S and G_2_-M (Fig. 7*A*; *n* = 3, *p* < 0.05).

To determine the effect of Ser^385^ in the location of the nuclear γ-tubulin C terminus, we transiently expressed Ser^385^-Cγ-tubGFP, Ala^385^-Cγ-tubGFP, or Asp^385^-Cγ-tubGFP in NIH3T3 cells (8). Immunofluorescence analysis showed a constitutive nuclear localization of these mutants (Fig. 7*B*), which supports the idea that the N terminus of γ-tubulin masks the NLS. However, Ala^385^-Cγ-tubGFP and Ser^385^-Cγ-tubGFP localized to the nucleus, the centrosomes, and the microtubules, but Asp^385^-Cγ-tubGFP was mostly in the nucleus and in the centrosomes (Fig. 7*B*). Although the presence of Asp^385^-Cγ-tubGFP was detected in single cells by immunofluorescence, neither anti-GFP nor anti-γ-tubulin antibodies detected the C-terminally tagged GFP Asp^385^-Cγ-tubulin mutant by immunostaining or Western blotting analysis (Fig. 7, *C* and *D*). Our findings indicate that the conformation of nuclear γ-tubulin differs from the cytosolic pool, in a similar way as described previously for nuclear actin (26).

Moreover, we found that the total cytosolic amount of Cγ-tubGFP increased by 16% when the Ala^385^-Cγ-tubGFP mutant was expressed (Fig. 7*C*), altogether suggesting that the cellular phosphorylation levels of Ser^385^-γ-tubulin play a role in determining the cellular localization of γ-tubulin.

##### Asp^385^-Cγ-tubGFP Is Not Associated with Microtubule Components

**FIGURE 8.**
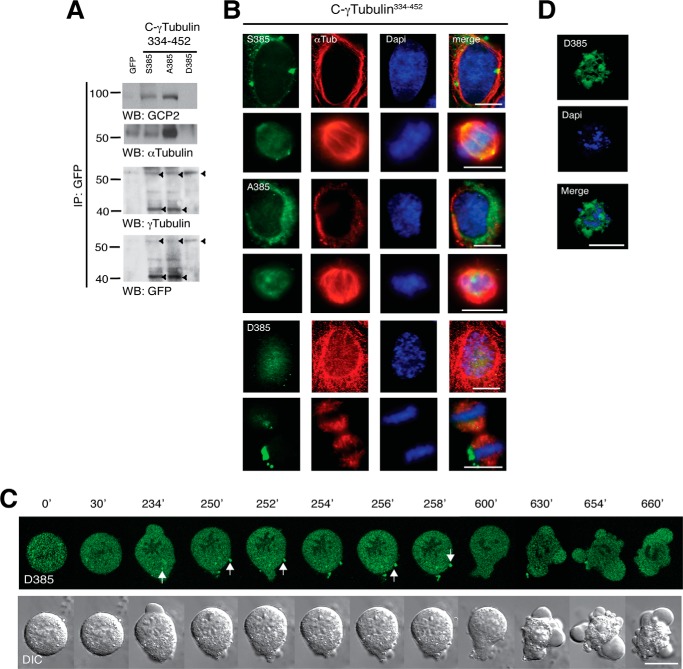
**Asp^385^-Cγ-tubGFP does not bind to GCP2 and α-tubulin and influence mitotic progression.** *A–D*, U2OS cells were simultaneously synchronized in early S phase as in Fig. 1*A* and transfected with GFP, Ser^385^-Cγ-tubGFP (*S385*), Ala^385^-Cγ-tubGFP (*A385*), or Asp^385^-Cγ-tubGFP (*D385*). *A*, Western blot shows the expression of the various Cγ-tubGFP mutants. The blots were analyzed with anti-GFP antibody and sequentially stripped and reprobed with antibodies against γ-tubulin, GCP2, and α-tubulin. *Arrowheads*, immunoprecipitated (*IP*) GFP-fused proteins (*n* = 3). *B*, after the double thymidine block treatment, U2OS cells expressing the indicated constructs were released for 9 h. Localization of the Cγ-tubGFP mutants was examined by immunofluorescence staining with anti-α-tubulin (α*Tub*; *red*) and nuclei DAPI staining (*blue*) in human U2OS cells incubated for 9 h (*n* = 3–5). *Scale bars*, 10 μm. *C*, differential interference contrast (*DIC*)/fluorescence images of time lapse from a U2OS cell with chromatin-bound Asp^385^-Cγ-tubGFP that arrests in metaphase. Images were collected every 2 min. The *image series* shows chosen frames of the location of Asp^385^-Cγ-tubGFP (*n* = 14). *D*, after double thymidine block treatment, U2OS cells expressing Asp^385^-Cγ-tubGFP (*D385*; *green*) were released for 24 h before being fixed. Nuclei were stained with DAPI (*blue*). Images show a representative dead U2OS cell that expresses Asp^385^-Cγ-tubGFP (*n* = 4).

In an attempt to achieve equal expression of the various Cγ-tubGFP mutants, we tested various transfections protocols. Upon simultaneous transfection and presynchronization of U2OS cells with thymidine (22), the various Cγ-tubGFP mutants were more evenly expressed (Figs. 7*C* and 8*A*). Western blot analysis of GFP immunoprecipitates with anti-γ-tubulin or -GFP antibodies detected two distinct bands. The expected relative molecular mass of Cγ-tubGFP is 43,200 Da, but the observed protein size varied from 43 to 60 kDa. Both Ser^385^-Cγ-tubGFP and Ala^385^-Cγ-tubGFP mutants were detected as a 43 and a 60 kDa band, whereas the phosphomimetic mutant, Asp^385^-Cγ-tubGFP, was only detected as a single 60 kDa band (Fig. 8*A*). In addition, analysis of the various Cγ-tubGFP immunoprecipitates disclosed a Ser^385^-dependent association with α-tubulin and GCP2 (Fig. 8*A*), which provides a potential explanation for the observed location of Ser^385^-Cγ-tubGFP and Ala^385^-Cγ-tubGFP in microtubules (Fig. 7*B*). In contrast, the phosphomimetic mutant, Asp^385^-Cγ-tubGFP, neither formed tubular structures nor associated with microtubule components (Fig. 7*B*), indicating that Ser^385^ regulates the binding of γ-tubulin to microtubules. However, both Ser^385^-Cγ-tubGFP and Ala^385^-Cγ-tubGFP are found in the nuclear compartment and are detected by Western blotting as a 60 kDa band, indicating that the conformation state of Asp^385^-Cγ-tubGFP can transiently be induced by environmental factors (27).

To ascertain whether the accumulation of cells in G_2_/M (Fig. 7*E*) is a consequence of mitotic failure due to an aberrant association of the Cγ-tubGFP mutants with mitotic chromosomes, we analyzed the effect of constitutive chromatin-bound γ-tubulin in mitotic cells by immunofluorescence studies (Fig. 8*B*). We found that upon mitosis entry, the Ser^385^-Cγ-tubGFP and the Ala^385^-Cγ-tubGFP mutant proteins were detached from the chromatin, whereas the Asp^385^-Cγ-tubGFP remained chromatin-bound during metaphase (Fig. 8, *B* and *C*); only cells with non-chromatin-bound Asp^385^-Cγ-tubGFP progressed through mitosis (supplemental Videos S1 and S2). However, cells with chromatin-bound Asp^385^-Cγ-tubGFP were unable to divide. The cells remained in metaphase during several h, and with time, the amount of chromatin-bound Asp^385^-Cγ-tubGFP decreased, and cytosolic aggregates were formed. Finally, cells formed blebs and died (Fig. 8, *C* and *D*; 28 ± 4%, *n* = 4). Mitotic cells expressing the Ser^385^-Cγ-tubGFP divided normally (supplemental Videos S3 and S4). Together, these results demonstrate that the cellular localization of γ-tubulin controls mitotic progression.

## DISCUSSION

Knowledge concerning the nuclear function of γ-tubulin is still limited, and one of the remaining questions is how cytosolic γ-tubulin becomes nuclear. To address this issue, we studied the presence of γTuRC components GCP2 and GCP3 and microtubules in different cellular compartments. In mammalian cell lines, we find neither GCP2, GCP3, nor α-tubulin in the nuclear fractions of the studied cells, suggesting a localization-dependent association of γ-tubulin with microtubules and γTuRC components. Moreover, we aimed to identify the mechanism responsible for import of γ-tubulin from the cytosol to the nucleus. Here, we found that the known microtubule (20) and centrosome (2) regulator, SadB, phosphorylates a serine residue near the γ-tubulin NLS (8). Indeed, despite the differences in the C-terminal regions of SadB_S_ and SadB_L_ (2) or in their cellular location, SADB_L_ and SadB_S_ phosphorylate γ-tubulin Ser^385^ and thereby govern the size of the nuclear pool of γ-tubulin. In support of that finding, overexpression of phosphomimetic mutants or SadB kinases increased the nuclear γ-tubulin pool. A decrease in SADB/SadB levels or expression of the non-phosphorylatable mutants reduced the nuclear pool of γ-tubulin. Also, the increase in nuclear γ-tubulin caused by overexpression of SadB_S_, γ-tubulin, or the phosphomimetic mutants increased the number of cells in S phase. Accordingly, S phase entry was delayed by decreases in levels of SadB (2) or γ-tubulin protein or expression of non-phosphorylatable mutants, and these effects were reverted upon introduction of the corresponding RNAi-resistant gene (2). Finally, in the absence of the N terminus, the Ser^385^-Cγ-tubGFP and Ala^385^-Cγ-tubGFP mutants localized to the centrosome, to the nucleus and to regions where microtubules are concentrated in comparison with the Asp^385^-Cγ-tubGFP that localized to centrosome and nucleus. These observations and the fact that SadB kinases and the Ser 385 motif are conserved among species strongly suggest that this phosphorylation plays a central role in the nuclear localization of γ-tubulin during cell division.

It has previously been shown that siRNA-mediated reduction of γ-tubulin levels inhibits centriole duplication and causes spindle defects in HeLa cells (28). Nonetheless, γ-*TUBULIN* shRNA- and γ-*tubulin* shRNA-transfected U2OS and NIH3T3 cells show no mitotic defects (2, 8, 9). An explanation for the differences between studies could be the limited reduction of γ-tubulin levels in cells transfected with shRNA (2, 8, 9). However, all γ-*TUBULIN* shRNA-induced defects reported by us are reverted upon introduction of an RNAi-resistant γ-*TUBULIN* gene (2, 8, 9) to exclude off-target effects caused by siRNA (29).

Microtubule formation requires longitudinal stabilization by nucleation onto γTuRC and a GTP-dependent conformational change of αβ-tubulin dimers (1, 4, 6). Although the domains and activities of β- and γ-tubulin GTPases are similar, the conformations of γ-tubulin-GDP and γ-tubulin-GTP are almost identical and resemble the curved depolymerized state of αβ-tubulins (1, 4, 6). Thus, phosphorylation of Ser^385^ may facilitate a conformational change that unmasks γ-tubulin's NLS and releases γ-tubulin from GCP2, GCP3, and microtubules. Several lines of evidence support this model. First, anti-total γ-tubulin and -GFP antibodies do not recognize the chromatin-bound phosphomimetic Ser^385^ → Asp γ-tubulin mutants in immunofluorescence studies, but the antibodies detect immunoprecipitates of Asp^385^-γ-tubulin and Asp^385^-Cγ-tubGFP by Western blotting. Anti-total-γ-tubulin recognizes Ser(P)^385^-γ-tubulin in membranes containing cell lysates with higher concentrations of the phosphorylated protein. Furthermore, we can with an anti-Ser(P)^385^-γ-tubulin antibody detect, in a phosphatase-dependent manner, endogenous levels of Ser(P)^385^-γ-tubulin or *in vitro* phosphorylated Ser^385^-γ-tubulin. Second, Ser(P)^385^-γ-tubulin and the γ-tubulin C terminus undergo a size shift in SDS gels. Finally, only Ser^385^-Cγ-tubGFP and Ala^385^-Cγ-tubGFP form tubular structures and associate with α-tubulin and GCP2. Altogether, the results reported here support the existence of different γ-tubulin conformational states that may aid γ-tubulin to bind structurally distinct proteins and in this way provide γ-tubulin with the observed functional properties (8, 10–14, 27).

Regarding the mechanism by which γ-tubulin regulates cell cycle progression, we think that the presence of nuclear γ-tubulin at early S phase turns off the mediated gene transcription of E2Fs (8, 9). Cell cycle progression is driven by the timely expression of cell cycle genes, such as cyclins (30). In most eukaryotes, there are three main waves of transcription, which coincide with the transition points G_1_-to-S, G_2_-to-M, and M-to-G_1_ (30). Interference with these transcription waves will inevitable disturb cell cycle progression as the expression of necessary genes is impeded. The transient phosphorylation of Ser^385^ at early S phase ends the first E2F-mediated transcriptional wave facilitating S phase execution (8), but the constitutive presence of nuclear Ser(P)^385^-γ-tubulin affects the mediated transcriptional waves of the following E2Fs (31) and thus cell cycle progression. This implies that the balance between chromatin-bound and microtubule-associated γ-tubulin may form a cellular sensor for transducing cytoskeletal alterations between compartments that can directly modulate gene expression during the cell cycle.

We propose that the fluctuating activities of SadB during G_1_ and S regulate the phosphorylation levels of γ-tubulin at Ser^131^. In this way, SadB enhances γ-tubulin polymerization at the nascent centriole and inhibits acentriolar formation of centrosomes elsewhere in the cell. However, phosphorylated Ser^131^-γ-tubulin also reduced astral microtubule nucleation at the centrosomes (2, 4), which probably facilitates the accessibility of SadB to Ser^385^ at the G_1_ to S phase transition. Phosphorylation of Ser^385^-γ-tubulin in the centrosomes then triggers a conformational change in γ-tubulin that releases this protein from GCP2, GCP3, and microtubules. In the nucleus, γ-tubulin puts an end to the activities of E2Fs, ensuring that the centrosomes and the chromosomes are replicated only once per cell cycle (2, 8, 16, 30).

Together, our data indicate that the transient increase in nuclear γ-tubulin during S phase is caused by SadB-mediated phosphorylation of Ser^385^, which induces a conformational change in γ-tubulin that leads to its nuclear accumulation during S phase. This identifies SadB as a multifunctional cell cycle regulator that triggers centrosome replication and S phase progression in mammals by controlling phosphorylation of Ser^131^ (2) and Ser^385^ in γ-tubulin.

## Supplementary Material

Supplemental Data

## Footnotes

2 The abbreviations used are:

NLS: nuclear localization signal
γ-tubGFP: human GFP-fused γ-tubulin
Cγ-tub: γ-tubulin C terminus
Nγ-tub: γ-tubulin N terminus
sh: short hairpin
E2F: E2 promoter-binding factor.
