# Supplementary material for: The Nuclear Localization of γ-Tubulin Is Regulated by SadB-mediated Phosphorylation
Source: J Biol Chem. 2014 Jun 18;289(31):21360–73. doi: 10.1074/jbc.M114.562389 (PMC4118101; doi:10.1074/jbc.M114.562389)
Supplement: Supplemental Data [file supp_289_31_21360__index.html]

The Nuclear Localization of γ-Tubulin Is Regulated by SadB-mediated Phosphorylation — The Nuclear Localization of γ-Tubulin — Supplemental Data 

# The Nuclear Localization of γ-Tubulin Is Regulated by SadB-mediated Phosphorylation

## Supplemental Data

**Files in this Data Supplement:**

- Supplemental video legends (.pdf, 64 KB) - Legends to the videos
- supplemental Video S1 (.avi, 3.8 MB)
- supplemental Video S2 (.avi, 2.0 MB)
- supplemental Video S3 (.avi, 2.1 MB)
- supplemental Video S4 (.avi, 1.3 MB)
