# Supplementary material for: The Nuclear Localization of γ-Tubulin Is Regulated by SadB-mediated Phosphorylation
Source: J Biol Chem. 2014 Jun 18;289(31):21360–73. doi: 10.1074/jbc.M114.562389 (PMC4118101; doi:10.1074/jbc.M114.562389)
Supplement: Supplemental Data [file supp_M114.562389_jbc.M114.562389-1.pdf]

The nuclear localization of γ-tubulin is regulated by SadB-mediated phosphorylation

Greta Eklund, Stefan Lang, Johan Glindre, Åsa Ehlén and Maria Alvarado-Kristensson

Supplemental Video

Supplemental Video S1. Illustrates DIC images of a mitotic U2OS cell expressing cytosolic D385-Cγ-tubGFP (*n* = 14, *n* represents the number of recorded mitotic cells).

Supplemental Video S2. Illustrates confocal images of the mitotic U2OS cell expressing cytosolic D385-Cγ-tubGFP (green) shown in Supplemental Video S1.

Supplemental Video S3. Illustrates DIC images of a mitotic U2OS cell expressing S385-Cγ-tubGFP (*n* = 6, *n* represents the number of recorded mitotic cells).

Supplemental Video S4. Illustrates confocal images of the mitotic U2OS cell expressing S385-Cγ-tubGFP (green) shown in Supplemental Video S3.
